# Taxonomic Re-Classification and Expansion of the Phylum Chloroflexota Based on over 5000 Genomes and Metagenome-Assembled Genomes

**DOI:** 10.3390/microorganisms11102612

**Published:** 2023-10-23

**Authors:** Sandra Wiegand, Morgan Sobol, Luca Kristina Schnepp-Pesch, Geng Yan, Sajid Iqbal, John Vollmers, Jochen A. Müller, Anne-Kristin Kaster

**Affiliations:** Institute for Biological Interfaces (IBG 5), Karlsruhe Institute of Technology, 76344 Eggenstein-Leopoldshafen, Germany; sandra.wiegand@kit.edu (S.W.); morgan.sobol@kit.edu (M.S.); luca.schnepp-pesch@gmx.de (L.K.S.-P.); gengyan2021@outlook.com (G.Y.); sajidiqbalmb44@yahoo.com (S.I.); john.vollmers@kit.edu (J.V.); jochen.mueller@kit.edu (J.A.M.)

**Keywords:** *Chloroflexota*, *Ca*. Dormibacterota, phylogenomics, microbial dark matter, big data science

## Abstract

The phylum *Chloroflexota* (formerly Chloroflexi) encompasses metabolically diverse bacteria that often have high prevalence in terrestrial and aquatic habitats, some even with biotechnological application. However, there is substantial disagreement in public databases which lineage should be considered a member of the phylum and at what taxonomic level. Here, we addressed these issues through extensive phylogenomic analyses. The analyses were based on a collection of >5000 Chloroflexota genomes and metagenome-assembled genomes (MAGs) from public databases, novel environmental sites, as well as newly generated MAGs from publicly available sequence reads via an improved binning approach incorporating covariance information. Based on calculated relative evolutionary divergence, we propose that *Candidatus* Dormibacterota should be listed as a class (i.e., *Ca*. Dormibacteria) within *Chloroflexota* together with the classes *Anaerolineae*, *Chloroflexia*, *Dehalococcoidia*, *Ktedonobacteria*, *Ca*. Limnocylindria, *Thermomicrobia*, and two other classes containing only uncultured members. All other *Chloroflexota* lineages previously listed at the class rank appear to be rather orders or families in the *Anaerolineae* and *Dehalococcoidia*, which contain the vast majority of genomes and exhibited the strongest phylogenetic radiation within the phylum. Furthermore, the study suggests that a common ecophysiological capability of members of the phylum is to successfully cope with low energy fluxes.

## 1. Introduction

Members of the bacterial phylum *Chloroflexota* occur in diverse environments including prevalence hotspots such as hot springs, wastewater treatment systems, and deep-sea sediments [1,2,3,4,5,6,7]. Culture-independent surveys have helped to expand known habitats of this phylum, e.g., members of the class *Anaerolineae* [8] predominate in the hypolimnion of deep lakes [9,10,11], indicating that they can have considerable importance in element cycling [12]. Members of the class *Dehalococcoidia* play an important role in the bioremediation of sites contaminated with chlorinated organic pollutants [13,14], while the class *Ktedonobacteria* [15] came into focus as a potential source of secondary metabolites that could be of medical relevance [16]. The metabolic capabilities in the phylum span the entire repertoire of principal microbial metabolic lifestyles, namely phototrophy, aerobic- and anaerobic respiration, fermentation, lithotrophy, organotrophy, heterotrophy, mixotrophy, and autotrophy. Metabolic diversity is already present on lower taxonomic levels, as shown by *Chloroflexus*, the eponymous type genus of the phylum and a member of the class *Chloroflexia* [17]. *Chloroflexus* spp. have been isolated from hot springs and are capable of anoxygenic phototrophy as well as aerobic respiration [18]. Given the trait heterogeneity within *Chloroflexota*, no defining phenotypic denominator of the phylum has been described, but thermophilic and neutrophilic growth as well as filamentous cell morphology have been frequently observed [7]. These examples illustrate the ecological and biotechnological significance of the phylum while also showing that its members exhibit great heterogeneity in terms of physiology and metabolism.

The taxonomic framework in which these traits evolved in the *Chloroflexota* is controversial. In phylogenetic tree topologies based on 16S rRNA gene sequence comparisons, the phylum is deeply branching [19,20]. However, recent phylogenomic analyses have indicated that the *Chloroflexota* could be phylogenetically much younger than originally thought [21,22,23]. Based on phylogenomics, the phylum is part of the “Terrabacteria” superphylum together with other monoderms [24,25,26], i.e., taxa lacking a lipopolysaccharide-containing outer membrane, atypical diderms, and potentially the candidate phyla radiation (CPR) [27]. The closest lineage considered to be next to the *Chloroflexota* is the *Candidatus* Dormibacterota, which comprises yet-uncultured ubiquitous terrestrial bacteria [28]. Their relative abundance increases in cold soils where they are apparently able to use atmospheric trace gases as electron donors for aerobic respiration [29]. Upon discovery, they were considered a candidate division (“AD3”) closely related to the *Chloroflexota* [30], then were placed as a sub-division into the *Chloroflexota* [31], and are currently classified as distinct candidate phylum [29].

Systematic classification within the *Chloroflexota* also greatly varies in taxonomic databases [19,32,33,34,35]. Validly published classes under the International Code of Nomenclature of Prokaryotes (ICNP) include the *Ardenticatenia* [36], *Caldilineae* [8], *Tepidiformia* [37], and *Thermoflexia* [38] in addition to the aforementioned *Anaerolineae*, *Chloroflexia*, *Dehalococcoidia*, and *Ktedonobacteria*. Furthermore, *Thermomicrobia* [39], *Ca*. Bathosphaeria, *Ca*. Limnocylindria (also named Ellin6529 [40], *Ca*. Edaphomicrobia [41], and RIF-CHLX [42]), *Ca*. Umbricyclopia [10], and *Ca*. Thermofontia (correct spelling of “*Ca*. Thermofonsia”, https://lpsn.dsmz.de/class/thermofontia, accessed on 14 July 2023) [43] are considered as class-level lineages within the *Chloroflexota* in some but not all taxonomic resources. In the genome taxonomy database (GTDB) [35,40], the phylum comprises 12 classes while the *Ca*. Dormibacterota are considered as a distinct sister phylum, whereas in SILVA [33], the *Chloroflexota* contain 17 classes—including the *Ca*. Dormibacteria—even after an extensive adoption of the GTDB taxonomy.

A major source of such discrepancies is differences in the phylogenetic trees calculated using either the 16S rRNA or genome sequences (whole or partial) ([33] and online note available at https://www.arb-silva.de/documentation/silva-taxonomy/, accessed on 14 July 2023). The differences can become more pronounced with greater sequence divergence, i.e., when comparing higher taxonomic levels. Furthermore, classification inconsistencies can be due to biases in taxon sampling and outgroup selection in phylogenetic tree computation [21]. Biases exist because genome-based phylogenies include a plethora of sequences from uncultured microorganisms. The inclusion of those has been almost discontinued in the curated database SILVA. Moreover, many metagenome-assembled genomes (MAGs) in GTDB do not contain rRNA genes and are therefore obviously not included in SILVA.

For a genome-based phylogeny, additional taxa sampling can be achieved through advanced mining of public databases to select and generate novel MAGs. In general, MAGs are constructed by assembling metagenomic sequences and binning the resulting contigs into consensus genomes which are each meant to represent a single, microbial taxon. Assembly is usually conducted with only one or a few datasets and samples. Respective contigs are then binned based primarily on nucleotide signatures, taxonomic markers, and contig coverage differences within each sample [44,45,46]. However, the most efficient binning processes also incorporate information on coverage covariance by using co-abundances across samples [44,45,47,48]. This approach provides much better resolution than relying only on information from a single sample; i.e., resolution improves when more related samples are present.

Here, we address taxonomic uncertainties of the *Chloroflexota* through a thorough phylogenomics analysis. To this end, we sampled 3456 publicly available genomes and metagenomes and assembled 1825 new medium-to-high-quality MAGs via improved binning and 76 MAGs from novel environmental samples. We then adopted GTDB’s quantitative criteria for taxonomic classification based on the computed relative evolutionary divergence (RED) values of selected marker proteins to make recommendations for classification updates. In addition to showing that a binning process based on multiple related samples allows for advanced MAG generation, we provide information on ecophysiological and cellular features of *Chloroflexota* classes including survival at low energy fluxes, catabolic substrates, and distribution of biosynthetic gene clusters and cell wall synthesis. Ultimately, extensive sequence analyses will foster our understanding of prokaryotes not better known than by their 16S rRNA gene sequence, i.e., microbial dark matter (MDM).

## 2. Materials and Methods

### 2.1. Public Genome and MAG Collection

**Figure 1:**
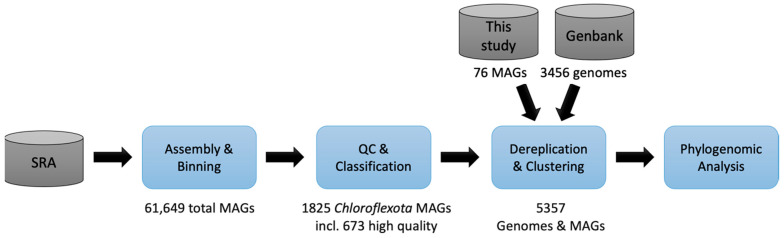
Workflow overview for re-classifying and expanding the taxonomy of *Chloroflexota*. Metagenomic datasets from pre-determined *Chloroflexota* habitats were downloaded from NCBI’s sequence read archive (SRA) database. Additional metagenome datasets from environmental samples were generated in this study. After metagenome assembly, binning, MAG classification, and quality control, a dataset consisting of 1825 MAGs from the SRA metagenomic data and 76 MAGs from the environmental data of this study was of intermediate or high quality and classified as *Chloroflexota*. An additional 3456 *Chloroflexota*, incl. *Ca*. Dormibacterota genomes from NCBI’s Genbank were downloaded in order to determine our MAG novelty and to create species clusters for phylogenomics analysis.

A sketch of the overall workflow is shown in Figure 1. Publicly available genomes of isolates and MAGs affiliated with *Chloroflexota* and *Ca*. Dormibacterota were downloaded in March 2022 from NCBI GenBank and PATRIC [49] if they were assigned to one of the following categories: “Chloroflexi”, their close relatives “Abditibacteriota”, “Armatimonadetes”, “Candidatus Eremiobacteraeota”, “Candidatus Dormibacteraeota”, “candidate division WS1”, “AD3” or “unclassified Terrabacteria group”. Furthermore, previously published MAGs [50] were downloaded through the IMG/M portal (https://img.jgi.doe.gov/, accessed on 1 April 2022) if they were classified as “Chloroflexota”, “Chloroflexota_A”, “Chloroflexota_B” or “Dormibacterota”.

The downloaded metagenomes were dereplicated to remove redundant or highly similar entries. Dereplication was carried out with dRep v3.2.2 [51] based on secondary FastANI clustering with a minimal overlap between genomes of 50%, a primary average nucleotide identity (ANI) threshold of 90%, and a secondary ANI threshold of 99% [52]. Genome quality was ignored during this step. Contamination and completion values were then determined as described in Section 2.5 “Quality processing, preliminary taxonomic classification, and clustering”. Only genomes that were categorized as at least “medium-quality draft” according to the MISAGs/MIMAGs standard [53] were used for further analyses. This filtering resulted in 3456 publicly available genomes (Supplementary Tables S1 and S2). Of these, 1162 were classified as high-quality (<5% contamination and >90% completeness), and the remainder were classified as medium-quality. The taxonomy of all genomes was also determined with the classify_wf workflow of GTDB-Tk v1.6.0 [54] to ensure compatibility with the novel MAGs and to exclude those not classified as *Chloroflexota* or *Ca*. Dormibacterota based on GTDB release 202 [35]. To include the phylogenetic context of these clades, additional representative genomes of “Terrabacteria” were downloaded in May 2022 with NCBI Entrez Programming Utilities (E-Utilities). The genomes were listed as representative of their species in GTDB release 202. FASTA files of amino acid sequences were created with Prodigal v2.6.3 [55].

### 2.2. Metagenome Dataset Selection, Categorization, and Downloading

Sequence Read Archive (SRA) metadata were accessed via the NIH data warehouse BigQuery on the Google Cloud platform using the STAT tool [56] to obtain a comprehensive overview of all metagenomic datasets that were found to harbor *Chloroflexota* or *Ca*. Dormibacterota reads. Query parameters are provided in the Supplementary Text. The percentages of *Archaea*, *Bacteria*, viruses/viroids, *Eukarya*, *Chloroflexota*/*Ca*. Dormibacterota, and unidentified sequences were then determined by dividing the total count per “name” entry by the total Illumina spot count. Ultimately, 235,575 SRA datasets with ≥10,000 total spot count, ≥5% Bacteria and an “organism” entry that contained “metagenome”, “enrichment culture”, “coculture” or “environmental sample” were used for analysis. The results of this habitat survey were then used to formulate NCBI E-utilities search terms for metagenomic datasets of potential interest (Supplementary Text). All metagenomes that appeared in more than one search category were deduplicated. SRA metadata for all found metagenomes were then gathered from BigQuery by SQL queries. Metagenomes that were not sequenced with an Illumina machine (to ease trimming) or had <1,000,000 reads were not considered for processing. Additionally, metadata were manually checked for plausibility. This approach left ten categories (bioreactors, corals and sponges, high salt, hot springs, lichens and mosses, marine, microbial mats, soil, water, and oral) from which metagenomes were selected for the assembly and binning process. Within each category, similar samples were grouped together for assembly, mostly depending on sampling spot and geographic characteristics. This was done only for similar samples to avoid excessive chimera formation. Binning groups were then formed to define the metagenomes that were all mapped to a specific assembly to allow binning with covariate coverage profile (Supplementary Table S2). In the “bioreactors, marine, water, and soil” categories, several data sets were processed individually to reduce computational costs. The metagenome datasets were then downloaded from SRA using prefetch v2.10.8 of the SRA toolkit (https://github.com/ncbi/sra-tools, accessed on 26 January 2022). Data were converted to fastq files with fastq-dump v2.10.8 of the SRA toolkit. All datasets were trimmed with Trimmomatic v0.39 [57], and paired-end reads (where applicable) were merged with FLASH v.1.2.11 [58].

Single amplified genomes (SAGs) of *Chloroflexota* were not included in the phylogenetic analyses since the majority of which has <50% completion, and exploratory tests with some medium quality SAGs showed that their inclusion would not modify tree topologies.

### 2.3. Metagenomes Obtained from Novel Environmental Samples

Additional metagenomes were generated from novel samples obtained from the following environmental sites: deep-sea sediment from Juan de Fuca Ridge in the Pacific Ocean off the coast of Canada; fumaroles and hot springs in the Azores; the Tatta Pani Hot Spring and Khewra Salt Mine, Pakistan; and four hot springs in Guangdong, China. More information on the sampling sites, including geographic coordinates and environmental parameters, is provided in the Supplementary Text.

Metagenomes were generated as previously described [59]. Briefly, genomic DNA was isolated from the samples using commercial kits with minor modifications. Quality and quantity of extracted DNA were determined with Nanodrop and Qubit spectrophotometers (Thermo Fisher Scientific Inc., Waltham, MA, USA). Metagenomic shotgun libraries of sheared DNA were prepared using the NEBNext^®^ UltraTM DNA Library Prep Kit (New England BioLabs, Frankfurt am Main, Germany). Libraries were sequenced on an Illumina NexSeq550 instrument (Illumina, San Diego, CA, USA).

### 2.4. Assembling, Mapping, and Binning

Assemblies were computed using MEGAHIT v1.2.9 [60] with all contigs >1500 bp and no *k*-mer multiplicity required. The rationale for the latter was to reduce the likelihood of chimeras (at the cost of more fragmented assemblies). In MEGAHIT, the standard approach of filtering *k*-mers below a specified multiplicity level helps to reduce “noise” introduced e.g., by sequencing errors or minor strain variants. Differences in *k*-mers that are present in some variants/reads but not others introduce new branches in the assembly graph, which usually results in contigs being broken into smaller parts. By setting a minimum *k*-mer multiplicity, it is possible to execute MEGAHIT in such a way that variants below a certain read coverage are ignored, thereby reducing the complexity of the graph and increasing the average contig lengths, but also reducing the sensitivity for strain variants and actually increasing the likelihood for potential chimeras. By modifying this parameter, the user can fine-tune the sensitivity for low abundant strain variants, ignoring only very low abundant strain variants that are hard to distinguish from sequencing errors. This setting is reasonable in most cases, especially if the research focus lies on more abundant taxa. Since we wanted to include low-abundant species in our analyses and have merged related samples from different sampling points or even studies, we chose not to ignore any *k*-mer variant in the assemblage graph, regardless of how low the coverage was. Rare variants were not merged with the corresponding majority variants, but assembled individually, resulting in more contig breaks, smaller average contig sizes, but higher sensitivity to strain variants or other very similar genome homologies. The *k*-mer lengths used started at 31 with increments of 10. The highest *k*-mer length was determined individually for each assembly based on the average length of the reads in all fastq files that were part of the assembly. In some cases, further steps were based on merged assemblies. Merging was accomplished with the dedupe.sh tool of the BBTools suite v38.79 (http://sourceforge.net/projects/bbmap/, accessed on 30 September 2022). Reverse complements were merged, requiring a minimal sequence identity of 99%.

To prepare the data for the binning, all metagenome datasets assigned to a binning group were mapped to the corresponding assembly. The untrimmed reads were mapped, and the coverage profiles were determined using the make and parse options of BamM v1.7.3 (https://github.com/ecogenomics/BamM, accessed on 30 September 2022).

To combine the strength and minimize the weaknesses of different binning approaches, binning was performed with three popular binning tools: CONCOCT v1.1.0 [44], MaxBin2 v2.2.7 [46], and MetaBAT 2 v2.12.1 [45] with default conditions using the recommended contig sizes of 1 kb (CONCOT and MaxBin2) and 1.5 kb (MetaBAT2). Subsequently, DAS Tool v1.1.2 [61] was used to integrate the three binning approaches at a score threshold of 0.1 and a duplicate penalty of 1, resulting in an optimized single set of bins. In some cases, the application of DAS Tool was too time-consuming due to the amount of data. Therefore, the most complete bins were selected with dRep v3.2.2 [51] based on secondary FastANI clustering [52] with a minimal overlap between the genomes of 50%, a primary ANI threshold of 90%, and a secondary ANI threshold of 99%. MIMAG genome quality was not considered at this early stage of the analysis.

### 2.5. Quality Processing, Preliminary Taxonomic Classification, and Clustering

All dereplicated MAGs from the binning approach and all *Chloroflexota* and *Ca*. Dormibacterota candidate MAGs from the environmental samples were analyzed with the classify_wf workflow of GTDB-Tk v1.6.0 based on GTDB release 202 to determine their preliminary taxonomy. Then, all MAGs classified as members of the *Chloroflexota*, *Ca*. Dormibacterota, and those unclassified at the phylum level were refined using the tool MDMcleaner v0.8.0 [62] to remove contigs that were incorrectly binned based on taxonomic evaluation of the individual contigs. Only contigs with the keep flag were used for further analysis. MAGs were re-evaluated with GTDB-Tk v1.6.0 to ensure their classification as *Chloroflexota* or *Ca*. Dormibacterota.

Quality in terms of completeness and contamination was determined to classify the MAGs based on the MISAGs/MIMAGs standard. To exclude chimeric genomes, contamination was determined by two methods and had to be <10% for both. CheckM [63] was used with the “taxonomy_wf” option. Contamination values were paralog-corrected (pc) as described previously [64] with the formula contamination_pc_ = contaminationCheckM − (contaminationCheckM × (strain heterogeneity/100)). Since contamination estimates of CheckM are constrained by its limited reference database, an additional contamination check was executed with MAGpurify [65] under default conditions, using contamination values determined based on the respective genome length. Completeness of MAGs was also determined by two different methods: CheckM was executed as described above and MDMcleaner was used with the “completeness” option enabled. The resulting completeness had to be >50% after weighting of the two results (completeness = 0.8 × CheckM + 0.2 × MDMcleaner).

All quality-validated genomes and MAGs from this study of at least intermediate quality were subjected to a thorough dereplication process to determine the novelty potential of the MAGs and whether a genome represents a species based on ANI threshold. This was again carried out using dRep v3.2.2 based on secondary ANImf clustering with minimal overlap between the genomes of 10%, a primary ANI threshold of 80%, and a secondary ANI threshold of 95%. It was also checked whether the best genome was publicly available or if a novel MAG was found in this study. The resulting clusters were classified into 11 groups according to the proportion of novel MAGs and their sources (Supplementary Text).

### 2.6. Annotations

All genomes selected as representatives of an identified species-level cluster were annotated using Prokka v1.14.5 [66] with the compliant, rfam, rnammer, and addgenes options enabled. The number of total genes, genes for coding (hypothetical) proteins, tmRNAs, tRNAs, rRNAs, and the analyses of gene length were based on these results. Additionally, the number of contigs, the N50, the L50, the genome size and the GC content were determined for each genome. The projected genome size was calculated by considering the weighted completeness determined above and extrapolating the genome size to 100% completeness. The genomes were also annotated using eggnog-mapper v2.1.8 to obtain COG (clusters of orthologous genes) and CAZy (catalytic and carbohydrate-binding modules) annotations [67]. The number and classes of biosynthetic gene clusters were determined by antiSMASH v5.1.2 run under default parameters for rapid execution [68]. Results were parsed from the gained gbk files.

To test for the presence of division and cell wall synthesis (*dcw*) genes in the *Dehalococcocidia*, profile HMMs that represent different aspects of peptidoglycan synthesis, cell division and elongasome formation were chosen: TIGR01072 (MurA), TIGR00179 (MurB), TIGR01082 (MurC), TIGR01087 (MurD), TIGR01085 (MurE), TIGR01143 (MurF), TIGR01133 (MurG), TIGR00445 (MraY), TIGR00904 (MreB), TIGR00219 (MreC), TIGR03426 (MreD), TIGR03423 (PBP2), TIGR01174 (FtsA), TIGR02673 (FtsE), TIGR00065 (FtsZ), TIGR02209 (FtsL), TIGR02223 (FtsN), TIGR02205 (ZipA), and TIGR02614 (FtsW). All medium-quality species-specific genomes were analyzed with hmmsearch implemented in HMMER v3.1b2. Furthermore, the *dcw* gene cluster from *Chloroflexus aurantiacus* J-10-fl was aligned against two high-quality genomes (when available) per order via MAUVE [69].

### 2.7. Phylogenomics Analyses

To compile a set of marker genes suitable for the clade “Terrabacteria” including the phylum *Chloroflexota*, 129 genes were initially tested (Supplementary Table S3). Profile HMMs for each gene were used to search the compiled collection of 20,942 genomes using hmmsearch implemented in HMMER v3.1b2 [70]. Individual cut-off values were used for each model (average of profile HMM parameters TC and NC). To account for fragmentation of the *rpoC* gene in Cyanobacteria, models TIGR02387 and TIGR02388 were used alongside TIGR02386 and concatenated when found. The same analyses were carried out on 2059 members of the PVC superphylum that served as the outgroup. After analyses of the positive hits according to hmmsearch, the marker gene set was reduced to 19 genes present in 10,141 “Terrabacteria” genomes plus 52 outgroup genomes. All hits from the profile HMM were individually aligned with MAFFT v7.505 [71] and the alignments were cleaned using trimAl v1.4.rev15 [72]. All alignments were then concatenated, and a phylogenetic tree was calculated with FastTree v2.1.10 [73] and rooted with the Biopython package Phylo. Analyses were also carried out at the level of one representative per species and one representative per genus. Additionally, balanced sampling was performed at the species and genus level by down-sampling overrepresented taxa. To accomplish this, taxa genome numbers were reduced to the number of genomes determined by 3 + (0.2 × (“genomes in taxa” − 3)). Taxa with three or fewer genomes were not reduced.

To analyze the phylogenetic relationship of *Chloroflexota* and *Ca*. Dormibacterota, a relative evolutionary divergence (RED) analysis was performed [40]. Calculation was carried out with PhyloRank v0.1.12 on the alignments mentioned above (https://github.com/dparks1134/PhyloRank, accessed in 30 September 2022). To compile a marker gene set suitable for phylogenomics of *Chloroflexota* and *Ca*. Dormibacterota, the same 129 genes were tested as described above. Profile HMMs for each gene were used to search those species-representing dereplicated genomes classified as high-quality (Supplementary Table S4). The marker gene set was then reduced to 50 genes that were present in 880 genomes plus 32 *Actinobacteria* outgroup genomes. Likewise, RED values were computed for the lineages *Tepidiformia* and *Tepidiformales*, *Ca*. Bathosphaeria (=UBA2979), *Ca*. Thermofontia, UBA2235, UBA4733, UBA5177, and UBA11872.

## 3. Results and Discussion

### 3.1. Novel Chloroflexota MAGs Assembled from Public Metagenome Datasets and Newly Sampled Habitats

First, we determined the relative abundances of nucleic acid sequences associated with *Chloroflexota* and *Ca*. Dormibacterota in publicly available metagenomic SRA datasets. The aim was to extend the standard metagenomic approach for single samples by grouping and combining multiple datasets. This approach maximizes information content and binning potential, allowing identification of corresponding sequences in datasets that were previously undetected. The analysis was not performed to determine relative taxa abundances in habitats analogous to 16S rRNA gene amplicon sequencing. The respective relative read abundances of the two approaches are not necessarily the same. The average relative abundance of sequences from the two taxa was highest in samples from hot springs (6.7%), followed by samples from microbial mats (0.8%) and metagenomes from decaying wood (0.7%). There were 70 datasets in which the respective relative sequence abundance ranged from 10% to over 70%, the majority of which were hot spring metagenomes. Metagenomic datasets were then grouped based on sampling sites and geographic characteristics to allow for greater variance in an assembly but also to ensure that the samples were principally compatible to avoid excessive chimera formation. Very large or unique datasets were not grouped prior to assembly but were processed individually to reduce computational burden and avoid chimera formation. In the case of related assembly groups, the assemblies were subsequently merged to increase variance and remove duplicate contigs. In a second step, reads from all metagenomic datasets used for each assembly were mapped to their respective contigs to obtain separate coverage information for each sample included in the final assembly (including merged assemblies). Furthermore, additional metagenomic datasets that were not directly part of the assembly (e.g., due to computational limitations) but were similar enough to be assigned to the same metagenome group were also mapped to obtain as much covariance information as possible for the binning processes.

Based on the results of the habitat analysis, we selected 866 metagenomic datasets that were processed in 242 individual assemblies and binned in 326 binning groups (Supplementary Table S5). Groups were further divided into ten environmental categories (bioreactors, corals and sponges, high salt, hot springs, lichens and mosses, marine, microbial mats, soil, water, oral). To maximize the resolution of the binning effort, three different binning tools were applied: MetaBAT 2 [45], MaxBin2 [46], and CONCOCT [44]. Results were integrated and de-replicated using the DAS Tool [60]. Through this approach, 61,649 MAGs were generated representing their corresponding binning group. After removing all MAGs <50,000 bp and those with a CheckM-determined contamination value of >20% or a completeness of <25%, and deduplication to 99% identity level, a total of 22,943 MAGs were used for further evaluation. While 8413 MAGs are derived from the “hot springs” category, the other habitat categories also add a substantial amount of data.

**Figure 2:**
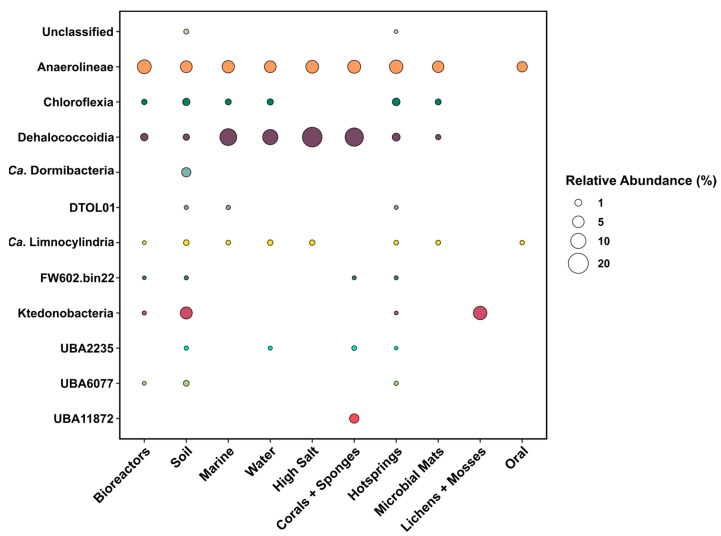
Abundances of MAGs affiliated with *Chloroflexota* and *Ca*. Dormibacterota in ten habitat categories relative to all MAGs from that category. Phylogenetic categorization is based on GTDB and thus differs from the phylogenetic tree shown in Figure 3. Color indicates the different classes. Absolute numbers of MAGs per category are provided in Table S6.

A GTDB-based taxonomic evaluation of all MAGs was used to identify the phylogenomic origins of the MAGs. *Chloroflexota* and *Ca*. Dormibacterota made up 3099 MAGs, while the remaining 19,844 MAGs were mostly *Proteobacteria*, *Bacteroidota,* and *Patescibacteria* (Supplementary Table S6). Most of the *Chloroflexota* MAGs were derived from the “hot springs” category (comprising 29.3% of all MAGs in that habitat category), followed by the categories “coral and sponges” (13.7%) and “high-salt” (13.4%) (Figure 2). A high recovery of *Chloroflexota* MAGs from these habitats has been described previously [74,75,76]. Most of the MAGs from the “hot springs”, “bioreactors”, “microbial mats” and “oral” categories are *Anaerolineae*, while most MAGs from the “marine”, “water”, “corals and sponges” categories belong to the *Dehalococcoides*. *Chloroflexia* MAGs are mostly derived from “hot springs” samples and *Ktedonobacteria* and *Ca*. Dormibacterota from “soil”.

Upon an additional refinement step via MDMcleaner [61], a total of 3047 MAGs were found to be members of the *Chloroflexota* or *Ca*. Dormibacterota (Table 1 and Supplementary Tables S7 and S8). Of these, 1825 MAGs had at least medium quality including, 673 with high quality according to the MISAG/MIMAG standard (>50% completeness and <10% contamination and >90% completeness and <5% contamination, respectively). These MAGs had between 2 and 3325 contigs, with a median of 388 (average of 471). Genome sizes were between 0.45 and 12.34 Mb, with a median length of 2.88 Mb (average 3.03 Mb). To determine the phylogenetic novelty of the 1825 MAGs from the binning approach and the 76 MAGs from the environmental samples sequenced in this study, 3456 publicly available *Chloroflexota* and *Ca*. Dormibacterota genomes of at least medium quality were added for further analyses. All 5357 genomes were clustered on the species level with an ANI of 95% [77], and the best genome was chosen as the representative. In total, 3508 species-level clusters were found, including 1055 represented only by novel MAGs from this study. The taxonomic knowledge for at least 201 clusters was broadened, as the best representative was a novel MAG and/or the majority of MAGs was novel. An initial analysis with GTDB-Tk of all MAGs with at least medium quality indicated 10 new orders in the *Anaerolinea* and 5 new orders in the *Dehalococcoidia*. We also performed the analysis with only high-quality MAGs (>90% completeness and <5% contamination). As shown in Table 1 and Supplementary Table S7, these include 1162 publicly available MAGs, 673 MAGs from our binning approach and 37 MAGs from the novel environmental samples. The approach resulted in 1485 species-level clusters, which is a 46% increase over the previous number of species-level clusters in the *Chloroflexota* and *Ca*. Dormibacterota. Based on the most stringent threshold of the GTDB-Tk analysis, the taxonomy of the phylum was extended by 3 new orders, 15 new families, 74 new genera, and 465 new species, all of which are represented by high-quality MAGs (Supplementary Tables S4–S7). Other MAGs would have further extended the taxonomy of other *Chloroflexota* classes but were disregarded because they were only of medium quality.

### 3.2. Ca. Dormibacteria as Class of Chloroflexota According to Relative Evolutionary Divergence

The phylogeny of *Chloroflexota* and their context in the whole “Terrabacteria” group are discussed controversially [29,31]. However, a common observation in several studies is that the phylum *Chloroflexota* and *Ca*. Dormibacterota appear to be monophyletic [22,28]. For additional genome-based testing on the monophyly of the two lineages, we constructed different phylogenetic trees of the “Terrabacteria” based on 19 marker genes present in at least 10,089 genomes representing a species within the clade. We carried out additional analyses with seven high-quality *Ca*. Dormibacterota genomes and with genomes representing not a species but a complete genus. Furthermore, we computed balanced trees that only featured even-numbered species or genera per overlying taxon. These tree-building approaches limit erroneous overemphasizing of taxa with many members in comparison with taxa with only few members. All of these phylogenetic analyses showed that the phyla *Chloroflexota* and *Ca*. Dormibacterota are monophyletic.

For hierarchical designation of *Chloroflexota* lineages, we computed relative evolutionary divergence (RED) values, which are the basis of taxonomic ranking in the GTDB [40]. RED values allow robust phylogenomic assessments based on thresholds derived from branch lengths, connecting parent nodes and the taxa they are comprising. For example, a RED value of 0.326 ± 0.1 is proposed to indicate the rank of phylum. When applied to our genome-based phylogeny, the *Ca*. Dormibacterota falls outside this interval, featuring a RED value of 0.527. When incorporated into the phylum *Chloroflexota* as a class-level taxon, the RED value of the phylum *Chloroflexota* is within the given interval before and after the incorporation while it changes from 0.329 to 0.285. According to this finding, we propose to classify *Ca*. Dormibacterota not as a phylum of its own but rather as the class *Ca*. Dormibacteria within the phylum *Chloroflexota*.

### 3.3. Chloroflexota Classes According to Genome-Based Phylogenetic Analysis

**Figure 3:**
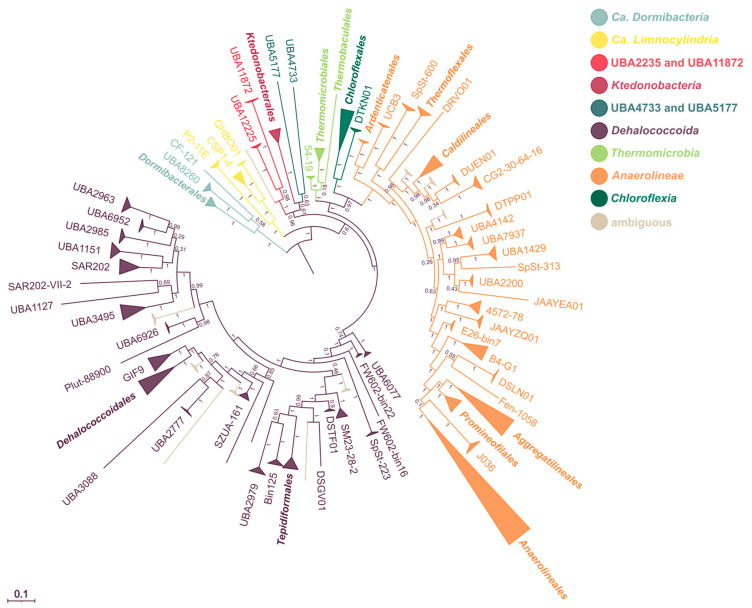
Phylogeny of the phylum *Chloroflexota* based on species representatives. Maximum likelihood phylogeny of the genome-based alignment from 50 concatenated protein marker genes present in 880 genomes and 32 outgroup genomes. Color indicates the different classes constituting the phylum. The individual leaf labels are on the order level. The node inscriptions give reliability estimators based on the Shimodaira–Hasegawa test.

To update the phylogenetic ranking within the *Chloroflexota* after the addition of the new MAGs, a phylogenetic tree based on 50 *Chloroflexota*-specific marker genes (Supplemental Table S4) present in at least 880 genomes representing a unique species was built (Figure 3). According to this tree and RED values, the phylum *Chloroflexota* contains the following seven classes with Latinized names—*Anaerolineae*, *Chloroflexia*, *Dehalococcoidia*, *Ca*. Dormibacteria, *Ktedonobacteria*, *Ca*. Limnocylindria, and *Thermomicrobia*—as well as two lineages comprised so far only of uncultured members, namely UBA2235/UBA11872 and UBA4733/UBA5177. Each of the latter lineages currently contains only few MAGs; therefore, we decided against selecting a representative sub-lineage (e.g., UBA2235 or UBA11872) and kept both names in the respective class designations. A single MAG (IMG ID 3300005529_81) could be a representative of another novel class, but since it is only of medium quality, we did not consider it further. The tree was tested by downscaling the dataset to include only genomes representing a complete genus and to only include a balanced number of species or genera per overlying taxon to avoid down-weighting of under-sampled taxa. In all instances, the *Aggregatilineales* (including *Ca*. Thermofontiaceae as family), *Ardenticatenales*, *Caldilineales*, and *Thermoflexales* branch deeply within the class *Anaerolineae*. RED values for these four clades are higher than 0.5, thereby supporting their placement as orders within the *Anaerolineae*. As with the *Ca*. Thermofontiaceae, the phototrophic *Chloroflexia* is an internal branch of the tree. This topology is consistent with evidence that anoxygenic phototrophy in that class is a trait acquired late rather than early in Earth’s evolutionary history [78].

Furthermore, when using 50 *Chloroflexota*-specific marker genes, the *Thermomicrobiales* and *Thermobaculales* did not cluster monophyletically within the class *Chloroflexia*. This pattern was also found when using fewer marker genes but was disrupted when the commonly used *rpoB* gene was solely used or when it was a dominant part of the underlying alignment. We therefore propose to avoid alignments with more than 20% of the amino acid sequence derived from *rpoB* for *Chloroflexota* datasets as the resulting trees show nodes not verified by trees built on more data. Furthermore, we suggest considering the orders *Thermomicrobiales*, *Thermobaculales,* and 54-19 as members of the standalone class Thermomicrobia, as seconded by an RED value of 0.35 for this lineage.

The order *Tepidiformales* branched within the *Dehalococcoidia* in all computed trees (RED value of 0.755), showing a closer phylogenetic relationship with this class than the neighboring classes *Anaerolineae* and *Chloroflexia* to each other. Therefore, we propose to merge the class *Tepidiformia* [37] with the *Dehalococcoidia*, keeping the latter as name for the class. Likewise, the *Ca*. Bathosphaeria and the *Ca*. Umbricyclopia [10] were lineages within the *Dehalococcoidia* in all computed trees (UBA2979 and members of Bin125 in Figure 3, respectively) and had RED values > 0.6, supporting their taxonomic classification as orders within *Dehalococcoida* rather than as distinct classes.

An interesting feature of the phylogenetic tree shown in Figure 3 is that the *Anaerolinea* and *Dehalococcoidia* appear to have undergone much greater phylogenetic radiation at the order level than the other classes of the *Chloroflexota*. This feature is mirrored in GTDB and SILVA, where the *Dehalococcoidia* have the third highest number of orders (50 in GTDB Release 207, 25 in SILVA Release 138) of all listed bacterial classes after *Gammaproteobacteria* (154/83) and *Alphaproteobacteria* (103/33). The number of orders in *Anaerolinea* (37/14) is also comparatively high in these databases. The cause of the apparent difference in radiation within the *Chloroflexota* is currently unknown. Sampling bias cannot be excluded but does not seem likely to be a main cause given the large number of publicly available metagenomes together with the bioinformatics capabilities to assemble MAGs representing novel orders in metagenomic datasets. This is not to say that we consider the biodiversity of *Chloroflexota* to be fully surveyed, but rather that the currently observable pattern of phylogenetic radiation may already represent a reasonable approximation of the intra-phylum’s macroevolutionary history. Furthermore, the *Dehalococcoidia* are less deeply branching than, e.g., the *Ktedonobacteria* in phylogenetic trees based on our analysis as well as on 16S rRNA sequence comparison [19], suggesting that the radiation pattern is not a mere result of past geological time. These considerations indicate that taxonomical coherence differs across the *Chloroflexota* classes.

### 3.4. Features of Chloroflexota Classes

Here we describe characteristics of genomes and MAGs (hereafter collectively referred to as “genomes” for short), including predicted traits of the classes not reported in detail elsewhere, along with a listing of typical habitats. The median projected genome size across all classes is 3.85 Mbp, and the average GC content is 57.6%. Predicted physiological traits (biosynthetic gene clusters, CAZy modules, COG categories) were plotted against genome size to identify features that are over- or under-represented in the genomes of a class compared to the whole phylum (Supplementary Figures S1–S14). Only over- or under-represented features are mentioned below.

Genomes affiliated with the *Anaerolineae* were the most abundant among the *Chloroflexota* in all samples (2518 genomes in total, Table 1) and were found in most habitat categories (Figure 2). Their genomes (median size: 3.56 Mbp, 56.6% GC) have slightly elevated relative numbers of glycoside hydrolase and glycosyltransferase genes than most other *Chloroflexota*, and the prevalence of giant genes (>5000 bp), which often encode surface proteins [79], was also comparably higher. These observations are consistent with the described growth of *Anaerolineae* in aggregates and biofilms, where they appear to be involved in anaerobic degradation of complex organic matter [7,80].

The 277 genomes belonging to *Chloroflexia* (median size: 5.07 Mbp, 61.7% GC) contain a comparatively high prevalence of giant genes similar to *Anaerolineae*. Furthermore, they tend to have a lower relative number of genes involved in amino acid transport and metabolism (COG category E). In their habitats, they have relative abundances around 1–3% based on MAG counts.

*Dehalococcoidia* have, on average, the smallest genomes across the phylum (median size: 1.81 Mbp, 55.0% GC). Genomes belonging to this class were the second-most abundant among the *Chloroflexota* in our analysis (1738 in total), with the highest prevalence in the habitat categories corals and sponges, high-salt, marine, and water. Many of their genomes harbor a higher proportion of genes involved in energy conversion (COG category C), partly due to the presence of multiple hydrogenase genes [81]. They also have a high relative abundance of genes involved in lipid transport and metabolism (COG category I). Fittingly, a high proportion of genes encoding enzymes for beta-oxidation were found in a *Dehalococcoidia* SAG from marine sediment of Aarhus Bay, Denmark [82]. The numbers of glycoside hydrolase and glycosyltransferase genes in *Dehalococcoidia* genomes are low, which corresponds to the comparatively low proportion of genes involved in central carbon metabolism in the core genome of *Dehalococcoides mccartyi* (4% versus e.g., 11% in *Escherichia coli*) [83]. Furthermore, *D. mccartyi* lacks the *dcw* gene cluster for division and cell wall synthesis, and no peptidoglycan layer was observed in electron microscopy or through staining in this microbe [14,81,84,85,86]. Instead, these bacteria possess a cell wall resembling the S-layer of *Archaea* [87]. In bacteria with a peptidoglycan layer, the *dcw* gene cluster is typically bordered on one side by two regulatory genes (*mraZ*, *mraW*) and on the other side by *ftsZ* and *ftsA*. These four genes are present and adjacent to each other in *D. mccartyi* and all investigated high-quality genomes of the *Dehalococcoidales* and the SAR202 cluster (two investigated genomes per order). This gene order arrangement is the same as in cell-wall-less *Mollicutes*, for which a loss of *dcw* genes during genome reduction is assumed [88]. In contrast, the *dcw* genes are present in the basal *Dehalococcoidia* lineages, such as the *Tepidiformales* and UBA6077. Apparently, a loss of *dcw* genes occurred in a common ancestor of the *Dehalococcoidales* and the SAR202 cluster. At least for *D. mccartyi*, it can be hypothesized that the replacement of the peptidoglycan layer by an S-layer-like cell wall results in lowering of fitness costs, which could be of particular importance for a microorganism with a limited flux of metabolic energy (see below).

*Ca*. Dormibacteria were found exclusively in soil samples (162 genomes). In all soil samples, they had relative abundances of approximately 1% of the total number of MAGs. Their genomes (median of 2.87 Mbp, 66.9% GC) showed a low prevalence of giant genes. Among the *Chloroflexota*, they have highest proportion of genes involved in amino acid transport and metabolism (COG category E) and, together with the *Dehalococcoidia*, of genes involved in lipid transport and metabolism (COG category I). Since proteins and lipids combined account for about 2/3 of the dry weight of a prokaryotic cell, these features indicate that *Ca*. Dormibacteria could thrive off decaying (microbial) biomass in their habitat [89] in addition to utilizing atmospheric trace gases [29].

The 128 genomes of the *Ktedonobacteria* are comparably large (median: 5.33 Mbp, 54.6% GC) and have more transposase genes in comparison, indicating a high level of genome plasticity as well as more transcription-related and rRNA genes. These features suggest that the members of this class adapt and evolve more readily to changing ambient conditions than the other *Chloroflexota*. Furthermore, they harbor larger numbers of identifiable biosynthetic gene clusters compared to the other classes, especially nonribosomal peptide synthetases/polyketide synthetases (NRPS/PKS) and clusters for ribosomally synthesized and post-translationally modified (RiPPs) lanthipeptide biosynthesis. The actin-like cytoskeletal encoding genes *mreBCD* were not found in any *Ktedonobacteria* genome. Their morphological analogs, the *Actinobacteria*, also do not harbor these genes [90]. Regarding habitat, they were found almost exclusively in soil, and lichens and mosses metagenomes, where their average relative abundance was 5.5% and 7.4% of all MAGs, respectively.

Genomes of *Ca*. Limnocylindria (282 genomes, 2.39 Mbp, 68.9% GC) and *Thermomicrobia* (141 genomes, 4.51 Mbp, 68.1% GC) had relative abundances around 1% in our data sets. Respective MAGs were found in all habitat categories except “corals and sponges” and “lichens and mosses” (Figure 2). To our knowledge, members of the *Ca*. Limnocylindria have so far only been known to occur in freshwater habitats, especially in deep lakes [10,91]. We identified no over- or under-represented COG category.

Genomes belonging to the proposed classes UBA4733 (3 genomes, 3.77 Mbp, 64.9% GC)/UBA5177 (9 genomes, 6.16 Mbp, 65.1% GC) and UBA2235 (50 genomes, 4.2 Mbp, 65.1% GC) had relative abundances < 1%. UBA5177 comprised, on average, the largest MAGs among the *Chloroflexota*. MAGs of UBA2235 had the highest relative proportion of genes from COG category G (carbohydrate transport and metabolism) among all *Chloroflexota*. MAGs of UBA11872 (48 genomes, 2.59 Mbp, 65.1% GC) were detected only in the category “corals and sponges”, where they were the third-most abundant *Chloroflexota* lineage at class level (10.6% of all *Chloroflexota* MAGs).

A literature survey suggests that features that seem to be shared by many *Chloroflexota* are the potential abilities to transform complex organic compounds and to survive at low energy fluxes. For example, members of SAR202 are abundant in the dark ocean, where they may be involved in the oxidation of recalcitrant organic matter [92,93,94], a capability they might share with *Ca*. Limnocylindria [10,91]. Similarly, members of *Dehalococcoidia* in deep-sea sediments apparently have a strictly anaerobic lifestyle involving homoacetogenesis together with resilience to decay, as suggested by analysis of several SAGs obtained from these habitats [4,6]. Niche specialization of organohalide-respiring *Dehalococcoidales* outside contaminated sites seems to be that they use naturally occurring organohalides with low abundance as electron acceptors [95]. Genome reduction in the *Dehalococcoidia* might be an adaptation to limited energy fluxes. It is a remarkable strategy. The small genomes of about 1.4 Mb of some *Dehalococcoidales* harbor only an incomplete suite of genes for cobalamin biosynthesis, although this is a co-factor of reductive dehalogenases essential in their catabolism [96]. Similarly, they have an incomplete Wood–Ljungdahl pathway and grow better in the presence of other microorganisms that complement their lacking capabilities [97]. The capability of other members of the phylum to successfully cope with low energy fluxes is illustrated by the abundant recovery of various aerobic *Chloroflexota* among very slow-growing soil bacteria [98], as well as the high prevalence of *Ca*. Dormibacteria in cold soils [28] and of CL500-11 (*Anaerolinea*) in the ultraoligotrophic Lake Michigan [9].

## 4. Conclusions

In this study, we have substantially expanded the genomic information of the *Chloroflexota* through a methodologically advanced generation of MAGs from related metagenomic datasets. The expanded MAG collection was used for phylogenetic analyses of the phylum, based on which we propose to list the *Ca*. Dormibacteria as class within the *Chloroflexota* phylum and make multiple suggestions for reclassification of lineages. According to RED values, the phylum contains the seven classes *Anaerolineae*, *Chloroflexia*, *Dehalococcoidia*, *Ca*. Dormibacteria, *Ktedonobacteria*, *Ca*. Limnocylindria, *Thermomicrobia*, UBA2235/UBA11872 and UBA4733/UBA5177. The *Ardenticatenia*, *Caldilineae*, and *Thermoflexia* are not classified as classes anymore but included as orders in the class *Anaerolineae*. The *Tepidiformales* are an order in the *Dehalococcoidia.* Furthermore, we show that *Anaerolineae* and *Dehalococcoidia* have undergone considerable phylogenetic radiation. As many lineages within the *Anaerolineae* and *Dehalococcoidia* are without cultured representatives, we are currently hampered in defining the niches in which radiation has taken place, i.e., there is only limited insight into their respective ecological interactions and biogeochemical interdependencies. An important limitation is the fact that metagenomics-based information will always carry a risk of being incomplete or contains chimeric MAGs despite thorough quality assessment. To minimize the risk of false conclusions, it is necessary to repeatedly re-evaluate publicly available MAGs, SAGs, and genomes of isolates to determine the currently most representative genome for each taxon at the species level. Furthermore, when analyzing the distribution of particular genome features, average gene counts across as many higher-level taxon representatives as possible (i.e., genus to class level) should be used to exclude misinterpretation due to assembly artefacts or single extreme outliers. However, it is hoped that the insights gathered from such cultivation-independent approaches will help to develop new targeted isolation and cultivation methods. Future elucidation of the evolutionary forces that led to phylogenetic and metabolic diversification will depend in no small part on knowledge of the in situ properties and functions of MDM in the *Chloroflexota*.

## Figures and Tables

**Table 1:** Numbers of genomes and MAGs per class analyzed in this study.

Class	Public ^1^ (All)	Public (h.q.) ^4^	Binning ^2^ (All)	Binning (h.q.)	Environ. ^3^ (All)	Environ. (h.q.)	Sum (All)	Sum (h.q.)
*Anaerolineae*	1522	563	968	367	28	11	2518	941
*Chloroflexia*	153	82	119	51	5	3	277	136
*Dehalococcoidia*	1100	331	605	207	23	10	1738	548
*Ca*. Dormibacteria	149	51	13	2	0	0	162	53
*Ktedonobacteria*	87	20	35	11	6	5	128	36
*Ca*. Limnocylindria ^5^	254	50	23	8	5	4	282	62
*Thermomicrobia* ^6^	112	39	22	12	7	4	141	55
UBA11872	13	7	35	11	0	0	48	18
UBA2235	43	15	5	4	2	1	50	20
UBA4733	3	2	0	0	0	0	3	2
UBA5177	9	2	0	0	0	0	9	2
IMG ID 3300005529_81	1	0	0	0	0	0	1	0
Sum	3456	1162	1825	673	76	38	5357	1873

^1^ Downloaded from public databases. ^2^ Binning = MAGs generated in this study by improved binning approach. ^3^ Environ. = MAGs generated in this study from novel environmental samples. ^4^ h.q. = high quality. ^5^ *Ca*. Limnocylindria were previously designated as class “Ellin6529” and are listed as such in preliminary MDMcleaner-based annotations (Supplementary Table S7; members: CSP1-4, P2-11E & QHBO01). ^6^ *Thermomicrobia* were previously designated as orders “Thermobaculales” and “Thermomicrobiales” as well as class “54-19” and are listed as such in preliminary MDMcleaner-based annotations (Supplementary Table S7).

## Data Availability

The sequence reads and MAGs generated in this study can be found in SRA, GenBank, and Zenodo as follows. All high- and moderate-quality *Chloroflexota* MAGs that are an improvement of the corresponding species cluster representation have been deposited at Zenodo under the following DOI: 10.5281/zenodo.7913253. Raw sequencing reads of metagenomes sequenced during this study have been deposited under the Bioprojects: PRJNA966135, PRJNA970198, PRJNA966133, and PRJNA901380. (The respective resulting high quality *Chloroflexota* MAGs that are an improvement of the corresponding species cluster representation have been uploaded under the respective Bioproject in addition to Zenodo (obligatory taxonomy check by NCBI curators is pending). High quality *Chloroflexota* MAGs that are an improvement of the corresponding species cluster representation that have been derived from reassembly of publicly available SRA datasets have been deposited under Bioproject PRJNA970559 in addition to Zenodo (obligatory taxonomy check by NCBI curators is pending).

## Supplementary Materials

The following supporting information can be downloaded at: https://www.mdpi.com/article/10.3390/microorganisms11102612/s1. Figure S1: Number of rRNA genes over genome length in different *Chloroflexota* classes; Figure S2: Number of giant genes over genome length in different *Chloroflexota* classes; Figure S3: Number of transposases over genome length in different *Chloroflexota* classes; Figure S4: COG category C (energy production and conversion) hits per genome over genome length in different *Chloroflexota* classes; Figure S5: COG category E (amino acid transport and metabolism) hits per genome over genome length in different *Chloroflexota* classes; Figure S6: COG category G (carbohydrate transport and metabolism) hits per genome over genome length in different *Chloroflexota* classes; Figure S7: COG category I (lipid transport and metabolism) hits per genome over genome length in different *Chloroflexota* classes; Figure S8: COG category K (transcription) hits per genome over genome length in different *Chloroflexota* classes; Figure S9: COG category M (cell wall/membrane/envelope biogenesis) hits per genome over genome length in different *Chloroflexota* classes; Figure S10: Average number of lanthipeptide biosynthetic gene clusters in *Chloroflexota* classes and orders; Figure S11: Average number of nonribosomal peptide synthetase/polyketide synthesase (NRPS/PKS) biosynthetic gene clusters in *Chloroflexota* classes and orders; Figure S12: Glycoside hydrolase genes per genome over genome length in different *Chloroflexota* classes.; Figure S13: Glycosyltransferase genes per genome over genome length in different Chloroflexota classes; Figure S14: Average occurrence of genes coding for enzymes involved in peptidoglycan biosynthesis (upper panel) or cell division and elongasome formation (lower panel) in *Dehalococcoidia* orders. Table S1: Public genomes; Table S2: Binning groups; Table S3: Markergenes; Table S4: Species clusters; Table S5: Overview MAGs; Table S6: Initial phylogeny MAGs; Table S7: Quality after MDMcleaner. Supplementary Text with information on: Determination of hotspots environments; Metagenome dataset selection and categorization; MAG clustering; Information on sampling sites of this study.
