# Supplementary material for: Taxonomic Re-Classification and Expansion of the Phylum Chloroflexota Based on over 5000 Genomes and Metagenome-Assembled Genomes
Source: Microorganisms. 2023 Oct 23;11(10):2612. doi: 10.3390/microorganisms11102612 (PMC10608941; doi:10.3390/microorganisms11102612)
Supplement: Supplementary file 1 [file microorganisms-11-02612-s001.zip › Supplementary_Text_and_Figures.pdf]

Supplementary Material

Taxonomic re-classification and expansion of the phylum *Chloroflexota* based on over 5,000 genomes and metagenome-assembled genomes

Sandra Wiegand[1], Morgan Sobol[1], Luca Kristina Schnepp-Pesch[1], Geng Yan[1], Sajid Iqbal[1], John Vollmers[1], Jochen A. Müller[1], Anne-Kristin Kaster[1*]

[1]Institute for Biological Interfaces (IBG-5), Karlsruhe Institute of Technology, Eggenstein-Leopoldshafen, Germany

*** Correspondence:**
anne-kristin.kaster@kit.edu

Supplementary Text

Determination of hotspots environments

SRA metadata were accessed in via the data warehouse BigQuery on the Google Cloud platform to get a comprehensive overview of all metagenomic datasets that were found to harbour Chloroflexota or Dormibacterota by STAT (https://genomebiology.biomedcentral.com/articles/10.1186/s13059-021-02490-0). The following information was gathered from the nih-sra-datastore:

(I) sra_tax_analysis_tool.tax_analysis_info.total_spot_count, sra_tax_analysis_tool.tax_analysis_info.analyzed_spot_count, sra_tax_analysis_tool.tax_analysis_info.unaligned_only, sra_tax_analysis_tool.tax_analysis.acc, sra_tax_analysis_tool.tax_analysis.tax_id, sra_tax_analysis_tool.tax_analysis.total_count, sra_tax_analysis_tool.tax_analysis.self_count, sra.metadata.acc, sra.metadata.organism in cases where sra_tax_analysis_tool.tax_analysis.rank was 'superkingdom', sra_tax_analysis_tool.tax_analysis.name was 'cellular organism', sra.metadata.assay_type was 'WGS' and sra.metadata.librarysource was 'METAGENOMIC'.

(II) sra_tax_analysis_tool.tax_analysis.acc, sra_tax_analysis_tool.tax_analysis.rank, sra_tax_analysis_tool.tax_analysis.name, sra_tax_analysis_tool.tax_analysis.total_count, sra_tax_analysis_tool.tax_analysis.self_count in cases where sra.metadata.assay_type was 'WGS', sra.metadata.librarysource was 'METAGENOMIC' and sra_tax_analysis_tool.tax_analysis.tax_id was 200795 (Chloroflexi) or 2052312 (Candidatus Dormibacteraeota).

Metagenome dataset selection and categorisation

NCBI Entrez was searched for metagenomic datasets of potential interest with E-utilities. The following search terms were used:

- "aquatic metagenome"[Organism] OR "aquifer metagenome"[Organism] OR "freshwater metagenome"[Organism] OR "freshwater sediment metagenome"[Organism] OR

"groundwater metagenome"[Organism] OR "lake water metagenome"[Organism] OR "pond metagenome"[Organism] OR "sediment metagenome"[Organism] OR "subsurface metagenome"[Organism]) AND WGS [All Fields]

- "activated sludge metagenome"[Organism] OR "anaerobic digester metagenome"[Organism] OR "biogas fermenter metagenome"[Organism] OR "bioreactor metagenome"[Organism] OR "bioreactor sludge metagenome"[Organism] OR "compost metagenome"[Organism] OR "decomposition metagenome"[Organism] OR "leaf litter metagenome"[Organism] OR "silage metagenome"[Organism] OR "sludge metagenome"[Organism] OR "wood decay metagenome"[Organism]) AND wgs[All Fields]
- "coal metagenome"[Organism] OR coal*[All Fields]) AND WGS [All Fields]'
- “coral metagenome"[Organism] OR "coral reef metagenome"[Organism] OR coral [All Fields] OR coral*[All Fields]) AND WGS [All Fields]
- earthworm [All Fields] AND WGS [All Fields]
- "hot springs metagenome"[Organism] OR hot OR thermal OR geothermal OR hydrothermal) AND WGS [All Fields]
- "hypersaline lake metagenome" [Organism] OR "saline spring metagenome" [Organism] OR "salt lake metagenome" [Organism] OR "salt marsh metagenome" [Organism] OR "salt mine metagenome" [Organism] OR "saltern metagenome" [Organism] OR salt [All Fields] OR saline [All Fields]) AND WGS [All Fields]
- "lichen metagenome"[Organism] OR lichen [All Fields]) AND WGS [All Fields]
- "microbial mat metagenome"[Organism] OR "stromatolite metagenome"[Organism]) AND wgs[All Fields]
- marine metagenome"[Organism] OR "hydrothermal vent metagenome"[Organism] OR "marine sediment metagenome"[Organism] OR"coal metagenome"[Organism] OR "deep sea"[All Fields]) AND wgs [All Fields]
- "moss metagenome"[Organism] OR moss [All Fields] OR moss* [All Fields]) AND WGS [All Fields]
- oral OR oral* OR (dent* calculus) OR (dent* AND calculcus) AND WGS [All Fields]
- "rice paddy metagenome"[Organism] OR paddy [All Fields] OR paddies [All Fields]) AND WGS[All Fields]
- "sponge metagenome"[Organism] OR sponge [All Fields] OR sponge* [All Fields]) AND WGS [All Fields]
- "soil metagenome"[Organism] OR "soil crust metagenome"[Organism]) AND WGS [All Fields])

Data reduction in the binning categories (*bioreactors*, *corals + sponges*, *high salt*, *hotsprings*, *lichens + mosses*, marine, *microbial mats, soil, water* and *oral*) were as follows.

- *Bioreactors*: Before the categories were defined, all metagenome datasets with less than <0.005% *Chloroflexota* (according to NCBI SRA) were removed. After the formation of the categories, only binning groups with at least >5% *Chloroflexota* in one comprised metagenome were kept. Additionally, those binning groups that were also part of a larger binning group (with >5% *Chloroflexota*) were kept.
- *Marine* and *water*: After the formation of the categories, only binning groups with at least >2% *Chloroflexota* in one comprised metagenome were kept. Additionally, those binning groups that were also part of a larger binning group (with >2% *Chloroflexota*) were kept.

- *Soil*: Before the categories were defined, all metagenome datasets with less than <0.001% *Chloroflexota* (according to NCBI SRA) were removed. After the formation of the categories, only binning groups with at least >0.75% *Chloroflexota* in one comprised metagenome were kept. Additionally, those binning groups that were also part of a larger binning group (with >0.75% *Chloroflexota*) were kept.
- No further data reduction was carried out for the 6 categories *corals + sponges*, *high salt*, *hotsprings*, *lichens + mosses*, *microbial mats* and *oral*.

MAG clustering

The MAG clusters were classified depending on the genomes they comprised as described below:

- *nothing_new_here*: no novel MAG was part of this species cluster
- *mostly_old*: <33% novel MAGs
- *old+new_onlyMAGsSRA*: 33-66% novel MAGs, all derived from the binning approach
- *old+new_onlyMAGsSample*: 33-66% novel MAGs, all derived from environmental samples
- *old+new_onlyMAGsSRA+Sample*: 33-66% novel MAGs, derived from the binning approach as well as environmental samples
- *mostly_new_onlyMAGsSRA*: >66% novel MAGs, all derived from the binning approach
- *mostly_new_onlyMAGsSample*: >66% novel MAGs, all derived from environmental samples
- *mostly_new_onlyMAGsSRA+Sample*: >66% novel MAGs, derived from the binning approach as well as environmental samples
- *new_onlyMAGsSRA*: only novel MAGs derived from the binning approach
- *new_onlyMAGsSample*: only novel MAGs derived from environmental samples
- *new_onlyMAGsSRA+Sample*: only novel MAGs, derived from the binning approach as well as environmental samples

Additionally, it was considered whether the best genome was publicly available or a novel MAG was found in this study.

Information on sampling sites of this study

Samples for novel metagenomes were collected from the following sites.

Two sites at the Juan de Fuca ridge flank (47°45′16.8″ N, 127°45′48″ W, 2667 meter water depth, 1.5-3 meters below sea floor, 2 °C, oxic, mud, August 2004; same site, 50-52 mbsf, 1.5-3 mbsf, 12 °C, anoxic, sandy silt; same site, 90-92 mbsf, 20 °C, anoxic, silt; 47°45′16.6″ N, 127°45′46.8″ W, 121-123 mbsf, 40 °C, anoxic, silt, August 2004).

Two sites on São Miguel, Azores (Pico Vermelho, 37°88′40.7″ N, 25°75′03.2″ W, pH 6, 52 °C, 2020; Calderiras Furnas, 37°77′46″ N, 25°30′59.8″ W, pH 4, 51 °C, 2020).

Tatta Pani Hot Spring, Pakistan (33°61′21.5″ N, 73°94′71.5″ E, pH 8.4, 62 °C, 2021)
Khewra Salt Mine, Pakistan (32°64′79.4″ N, 73°00′83.9″ E, pH 7.25, 24 °C, 2021)

Four sites in Guangdong, China (22°09′50″ N, 112°37′00″ E, pH 7.84, 35 °C, January 2021; 22°09′10″ N, 112°36′40″ E, pH 7.83, 55 °C, January 2021; 24°52′24″ N, 114°07′10″ E, pH 6.92, 60 °C, December 2020; 24°42′55″ N, 113°44′20″ E, pH 6.15, 65 °C, December 2020)

Supplementary Figures

For Figures S1-S14, the colours of the dots, bars and regression lines represent the class of the genome. Genome length is given in megabase pairs. In total, 3509 species-representing genomes were analysed.

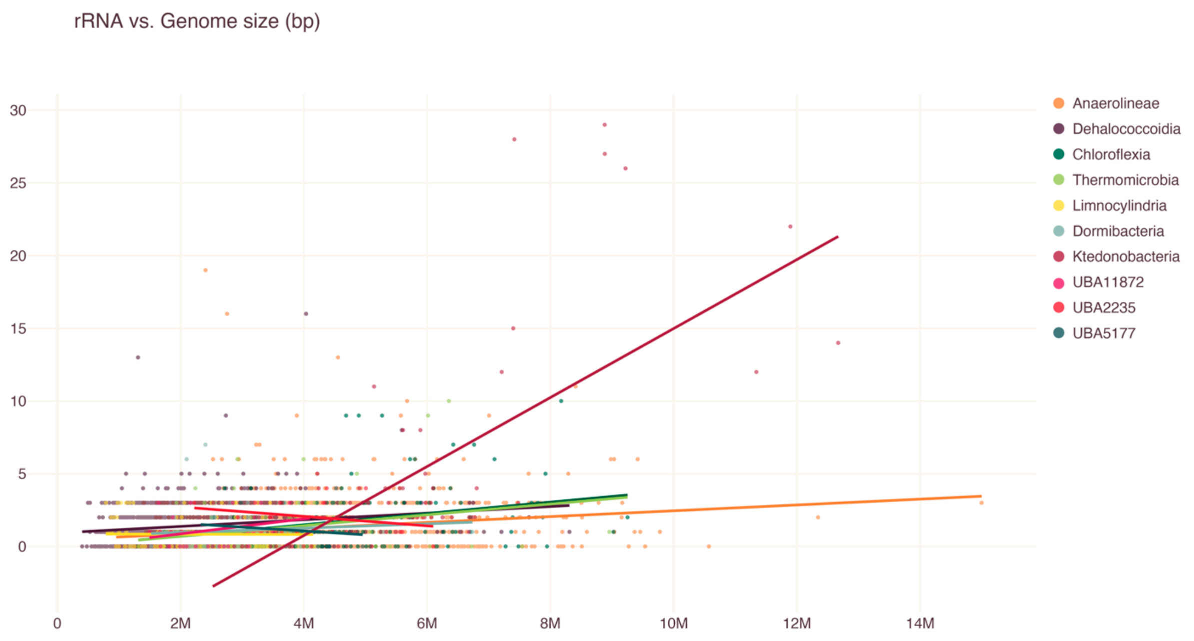

Supplemental Figure S1. Number of rRNA genes over genome length in different *Chloroflexota* classes.

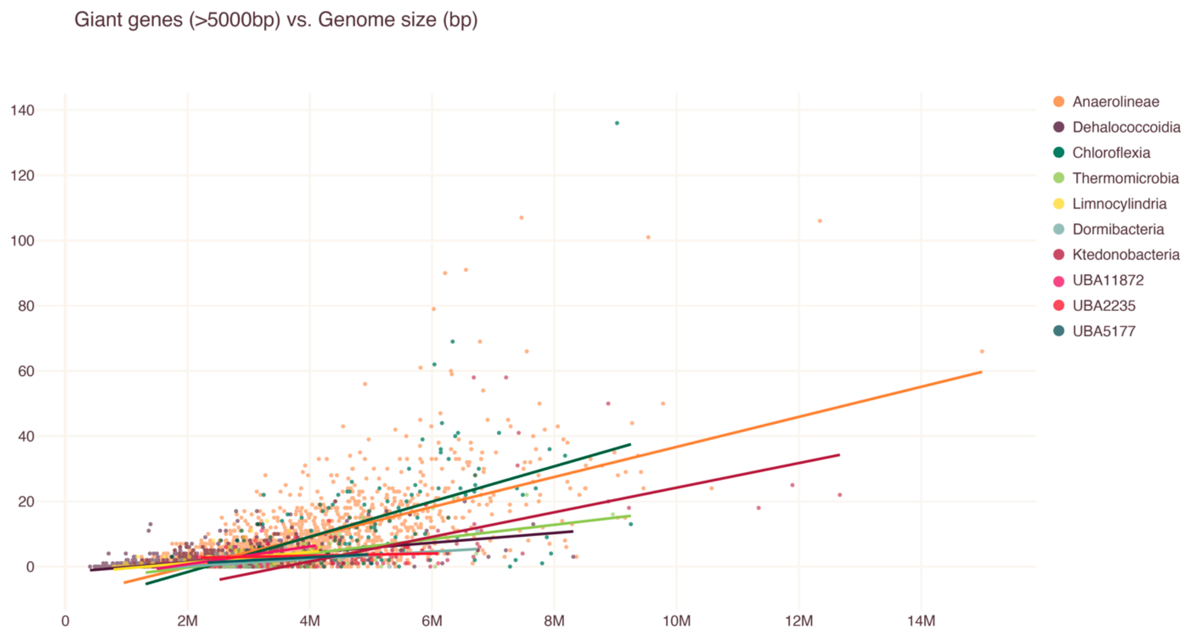

Supplemental Figure S2. Number of giant genes over genome length in different *Chloroflexota* classes.

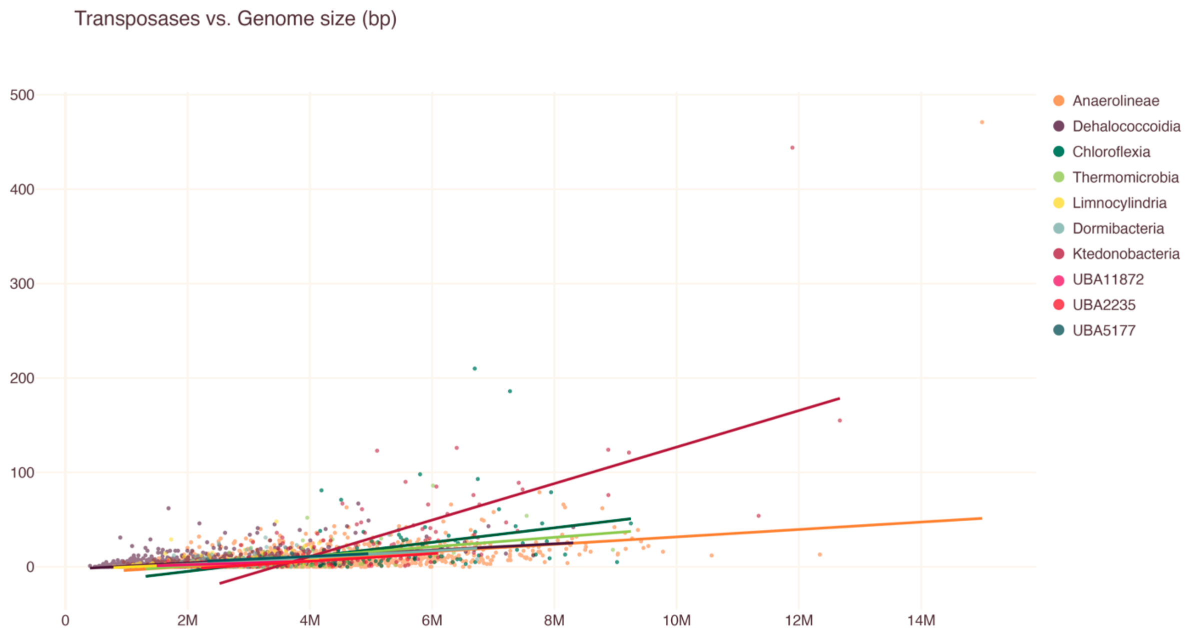

Supplemental Figure S3. Number of transposases over genome length in different *Chloroflexota* classes.

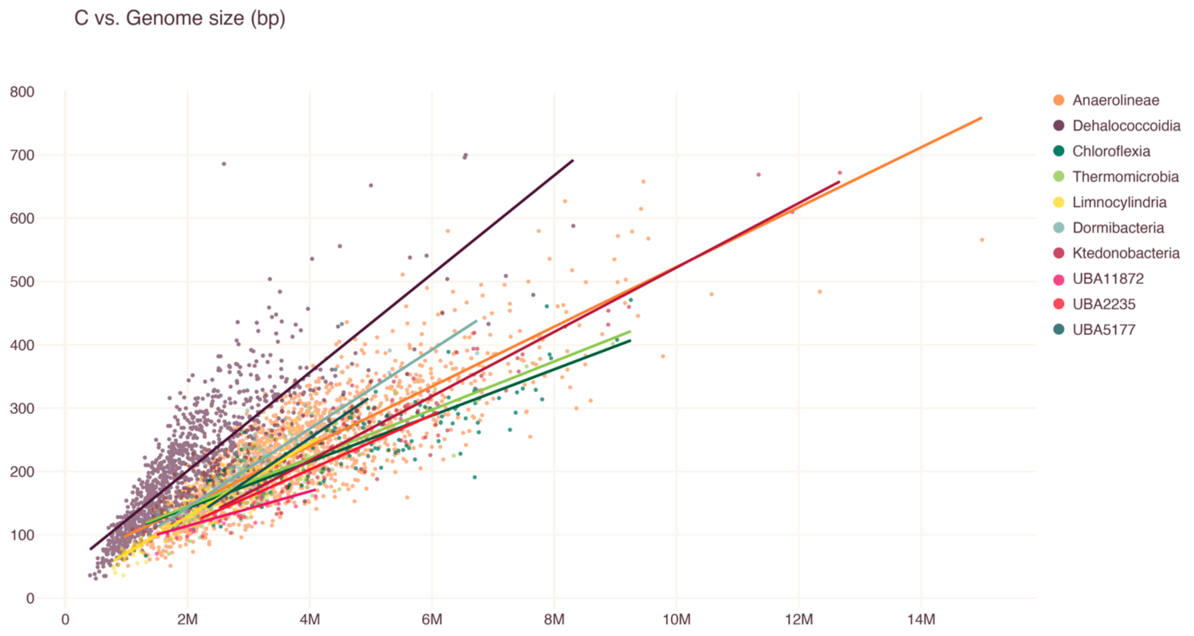

Supplemental Figure S4. COG category C (energy production and conversion) hits per genome over genome length in different *Chloroflexota* classes.

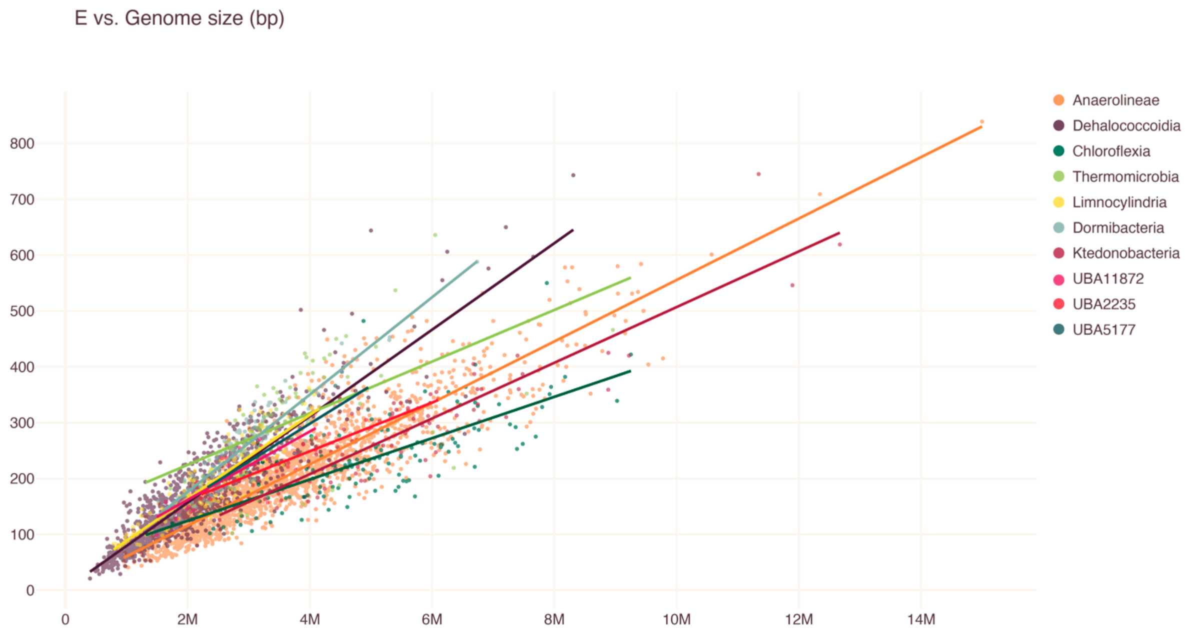

Supplemental Figure S5. COG category E (amino acid transport and metabolism) hits per genome over genome length in different *Chloroflexota* classes.

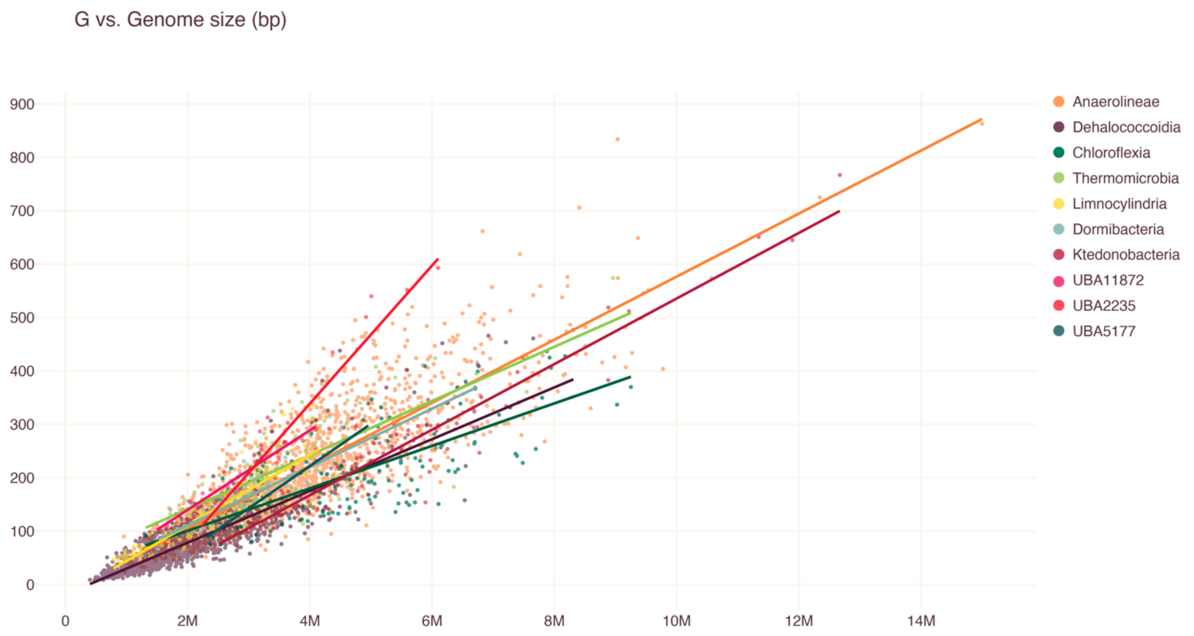

Supplemental Figure S6. COG category G (carbohydrate transport and metabolism) hits per genome over genome length in different *Chloroflexota* classes.

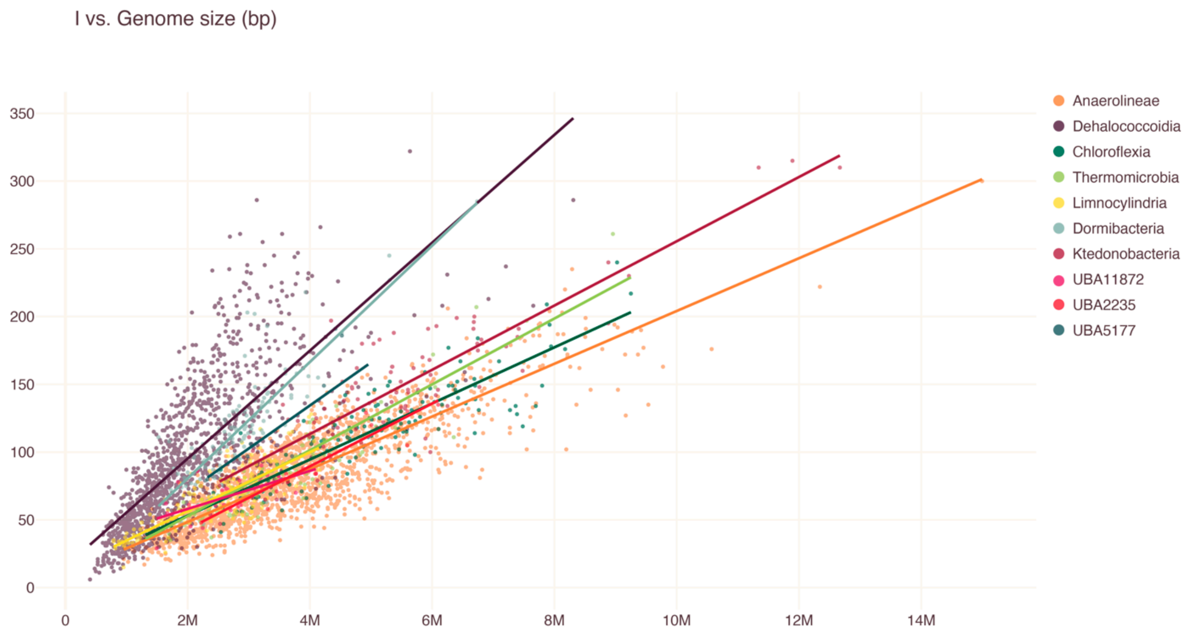

Supplemental Figure S7. COG category I (lipid transport and metabolism) hits per genome over genome length in different *Chloroflexota* classes.

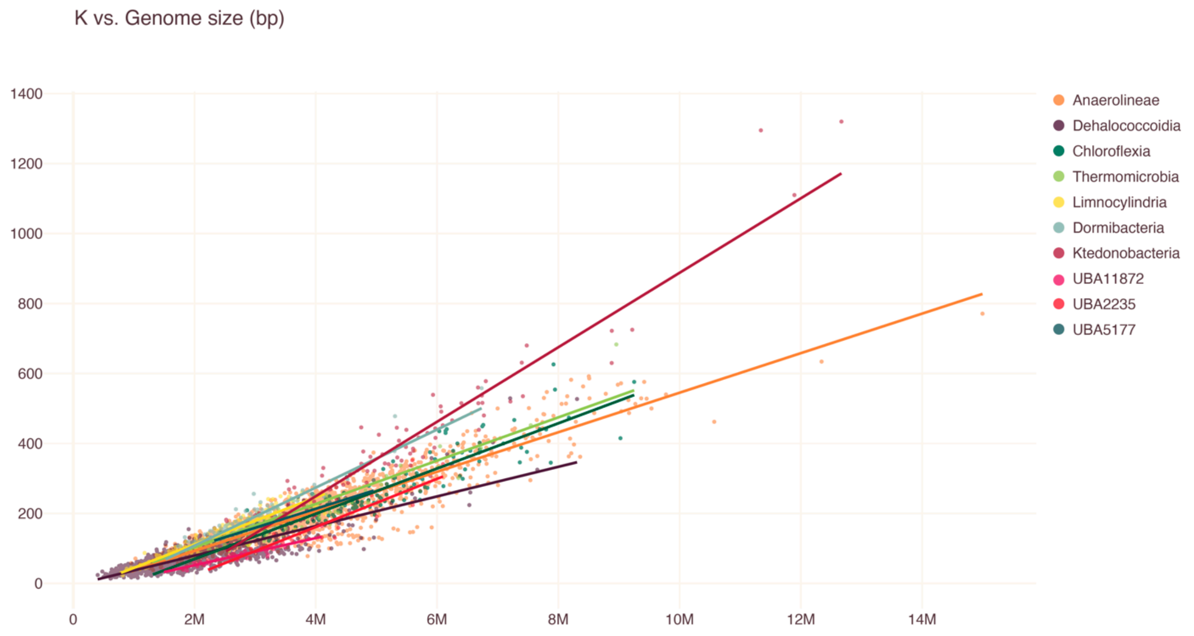

Supplemental Figure S8. COG category K (transcription) hits per genome over genome length in different *Chloroflexota* classes.

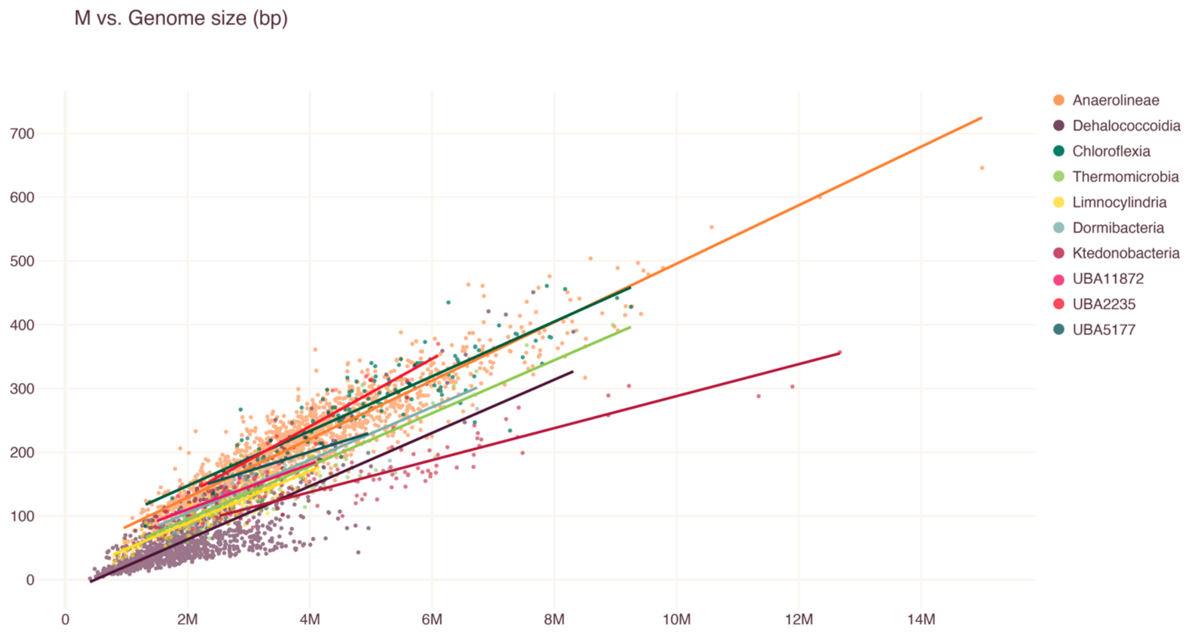

Supplemental Figure S9. COG category M (cell wall/membrane/envelope biogenesis) hits per genome over genome length in different *Chloroflexota* classes.

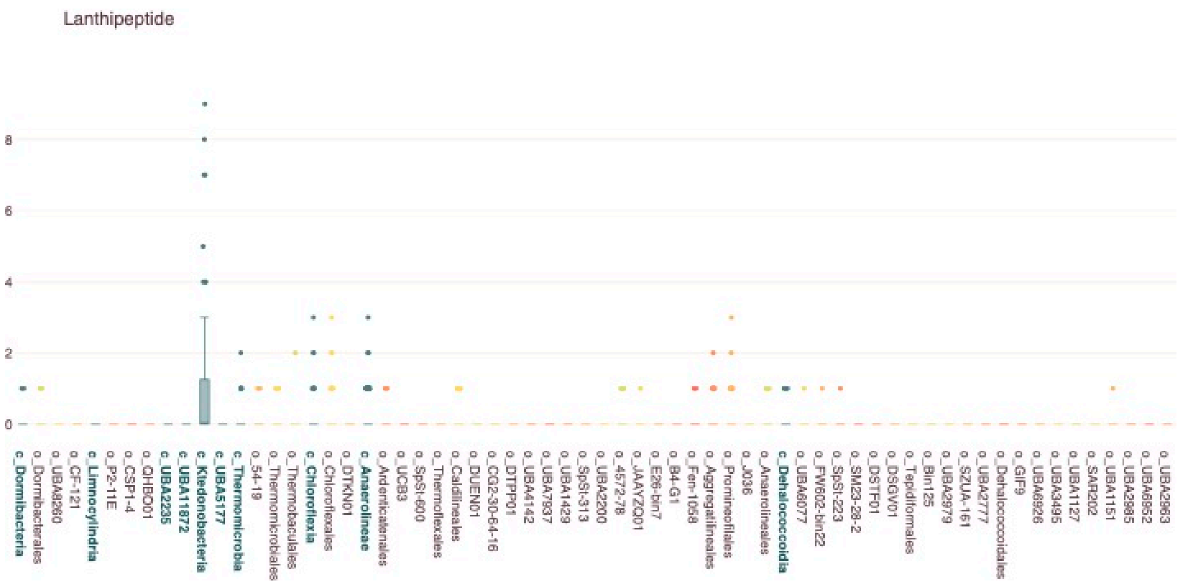

Supplemental Figure S10. Average number of lanthipeptide biosynthetic gene clusters in *Chloroflexota* classes and orders. Greyish blue boxes represent classes and yellow to red boxes represent underlying orders. Orders were included when there were at least two orders with 5 or more genomes in the encompassing class.

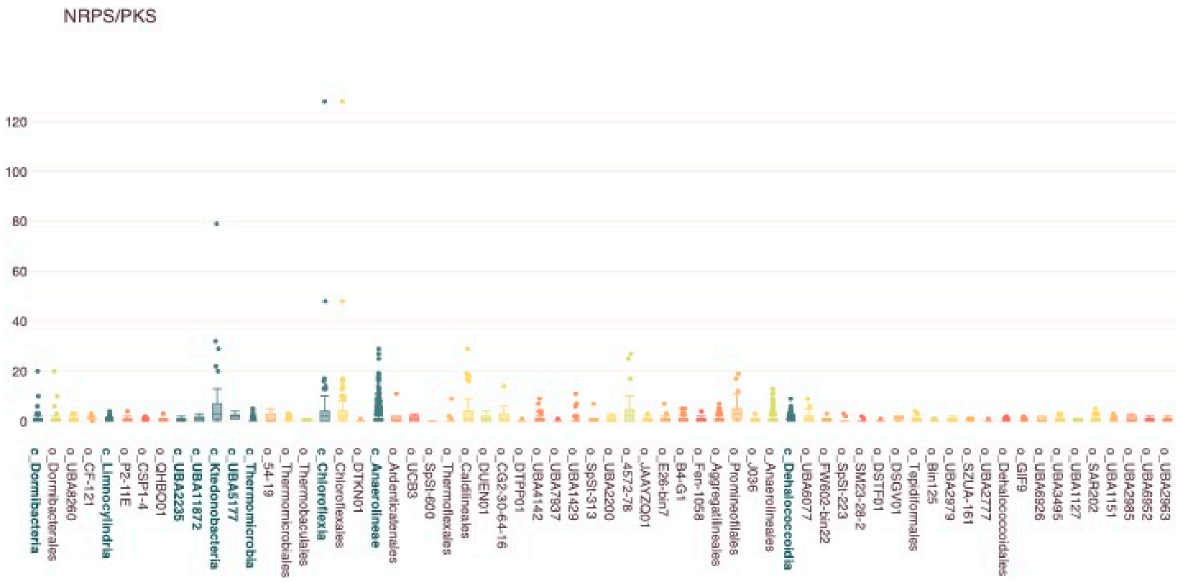

Supplemental Figure S11. Average number of nonribosomal peptide synthetase/polyketide synthesase (NRPS/PKS) biosynthetic gene clusters in *Chloroflexota* classes and orders. Greyish blue boxes represent classes and yellow to red boxes represent underlying orders. Orders were included when there were at least two orders with 5 or more genomes in the encompassing class.

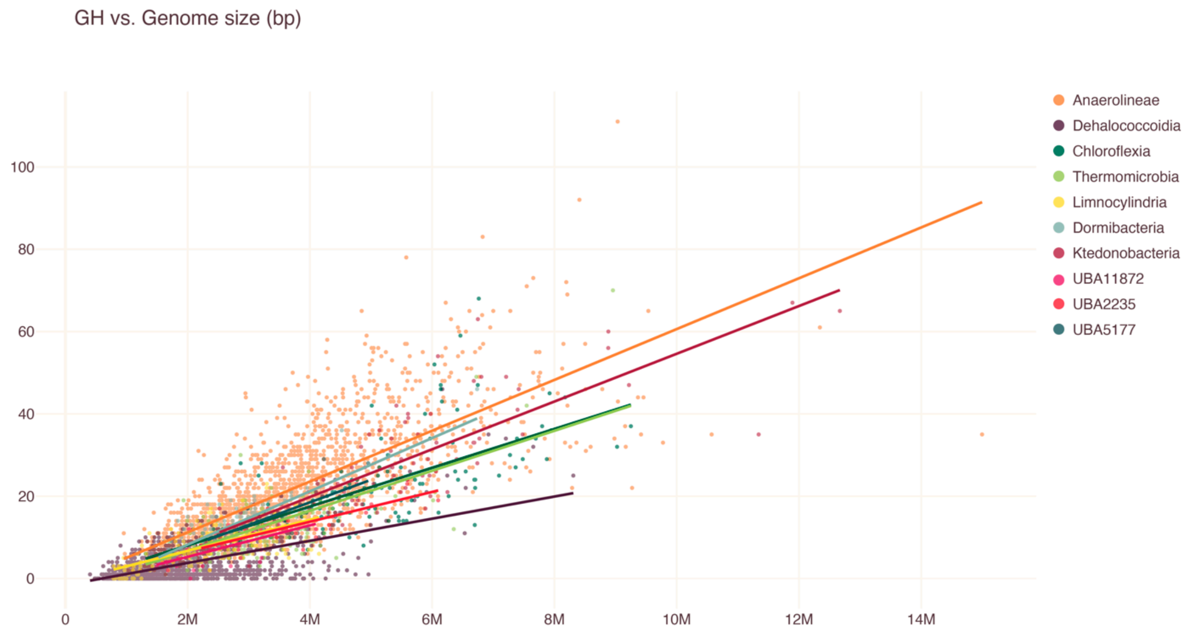

Supplemental Figure S12. Glycoside hydrolase genes per genome over genome length in different *Chloroflexota* classes.

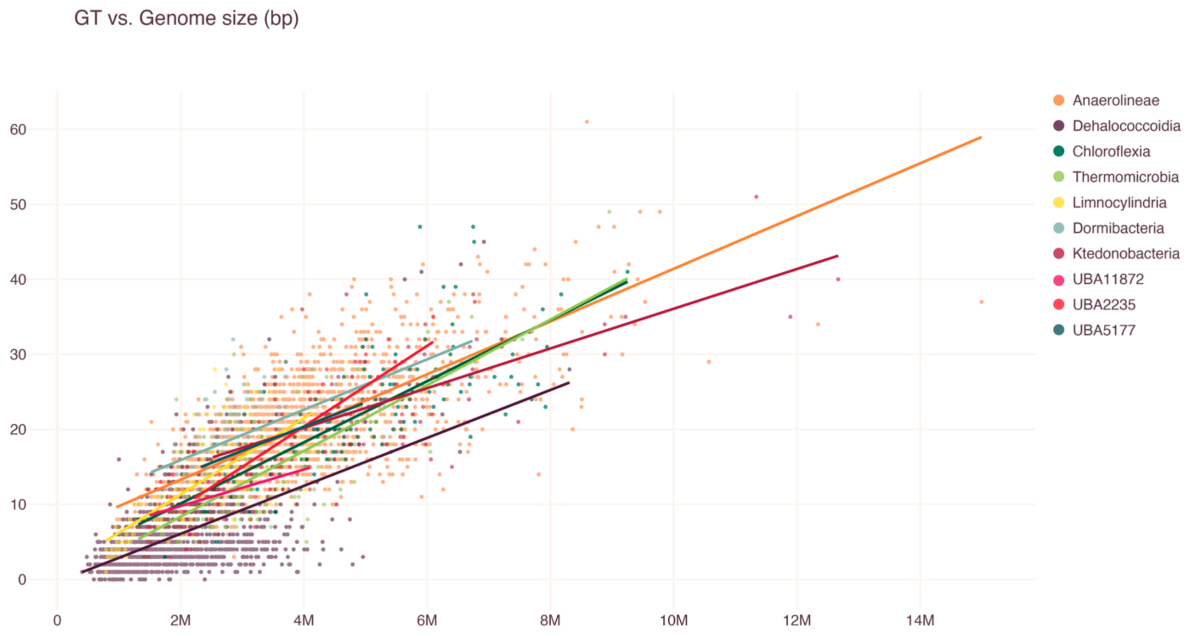

Supplemental Figure S13. Glycosyltransferase genes per genome over genome length in different *Chloroflexota* classes.

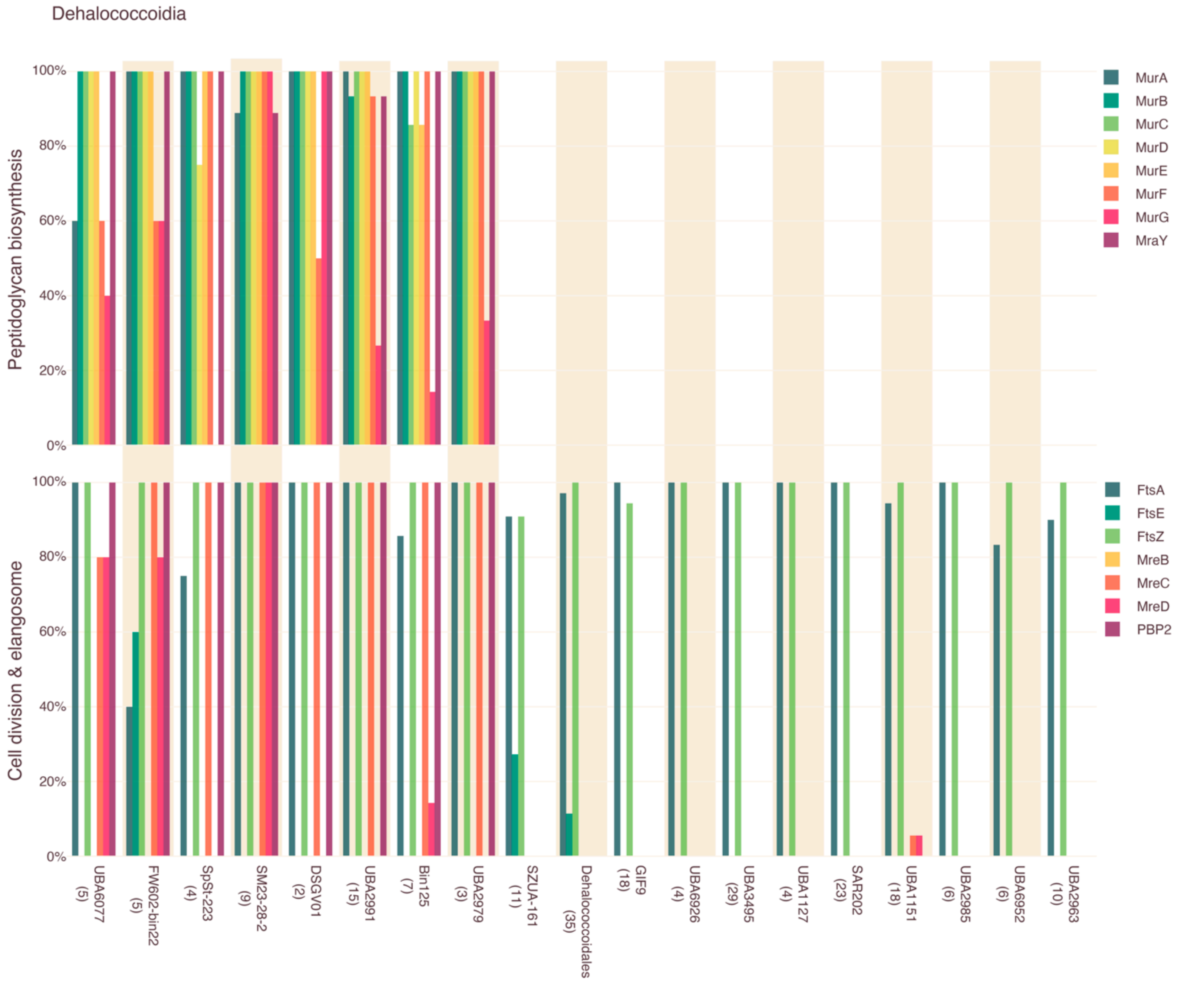

Supplemental Figure S14. Average occurrence of genes coding for enzymes involved in peptidoglycan biosynthesis (upper panel) or cell division and elongasome formation (lower panel) in *Dehalococcoidia* orders. Numbers indicate the number of genomes analysed for each order. The results for FtsL, FtsN, ZipA and FtsW are not shown as they either had hits in less than 1% percent of all genomes or had multiple inconclusive hits in most genomes.

1.1 Supplementary Tables

Supplementary Tables S1-S7 are uploaded as individual Excel files
